# The Impact of COVID-19 Lockdowns in Germany on Mood, Attention Control, Immune Fitness, and Quality of Life of Young Adults with Self-Reported Impaired Wound Healing

**DOI:** 10.3390/jcm12093205

**Published:** 2023-04-29

**Authors:** Jessica Balikji, Anna H. Koyun, Pauline A. Hendriksen, Pantea Kiani, Ann-Kathrin Stock, Johan Garssen, Maarten M. Hoogbergen, Joris C. Verster

**Affiliations:** 1 Division of Pharmacology, Utrecht Institute for Pharmaceutical Sciences, Utrecht University, 3584 CG Utrecht, The Netherlands; 2 Cognitive Neurophysiology Department of Child and Adolescent Psychiatry, Faculty of Medicine of the TU Dresden, University of Dresden, D-01307 Dresden, Germany; 3 Biopsychology, Department of Psychology, School of Science, TU Dresden, 01062 Dresden, Germany; 4 Global Centre of Excellence Immunology, Nutricia Danone Research, 3584 CT Utrecht, The Netherlands; 5 Division of Plastic Surgery, Catharina Ziekenhuis, 5623 EJ Eindhoven, The Netherlands; 6 Centre for Human Psychopharmacology, Swinburne University, Melbourne, VIC 3122, Australia

**Keywords:** mood, attention, attention focusing, attention shifting, impaired wound healing, slow healing wounds, wound infection, immune fitness, quality of life

## Abstract

Background: Previous studies in Dutch young adults revealed that individuals with self-reported impaired wound healing reported poorer mood, increased inattention and impulsivity, poorer quality of life, and poorer immune fitness compared to healthy controls. Another study revealed that the negative impact of lockdowns during the 2019 coronavirus disease (COVID-19) pandemic was significantly more profound among the impaired wound healing group than the control group. The purpose of the current study was to replicate and extend these findings among young adults living in Germany. Methods: A retrospective, cross-sectional survey was conducted among N = 317 young adults living in Germany, 18–35 years old. They were allocated to the IWH group (N = 66) or the control group (N-251). Participants completed the Attention Control Scale, and mood, quality of life, and immune fitness were assessed with single-item ratings. All assessments were made for (1) the period before the COVID-19 pandemic, (2) the first lockdown period, March–May 2020, (3) the first no-lockdown period, summer 2020, (4) the second lockdown, November 2020 to May 2021, and (5) the second no-lockdown period, summer 2021. Results: The impaired wound healing group reported significantly poorer mood, quality of life, and immune fitness. The effects were evident before the pandemic. The impaired wound healing group scored significantly poorer on attention focusing, but no significant differences between the groups were found for attention shifting. During the pandemic, negative lockdown effects (i.e., further aggravation of mood and immune fitness and lower quality of life) were evident in both groups but significantly more profound in the impaired wound healing group. No differences between the groups were found for the no-lockdown periods. Conclusion: Individuals with self-reported impaired wound healing have significantly poorer mood, attention focusing, and immune fitness and report a poorer quality of life than healthy controls. The impact of COVID-19 lockdowns was significantly more profound in the impaired wound-healing group.

## 1. Introduction

The lockdown periods during the 2019 coronavirus disease (COVID-19) pandemic had substantial negative socioeconomic consequences [1,2].

Although data showed that the impact of lockdowns is highly heterogeneous, depending on the sample under investigation, for individuals who struggled to cope with lockdown restrictions, these periods have been associated with poorer mood (e.g., anxiety, depression, loneliness, and stress) [3,4,5,6]. As a consequence of the lockdown restrictions, delayed healthcare was also common [7,8]. Hospital referrals were canceled or postponed, which led to delayed diagnosis and treatment for acute or chronically ill patients, sometimes with demonstrated negative health consequences such as an increased burden of disease [7] and reduced functional outcomes [8].

In Germany, several actions were taken to limit the spread of the SARS-CoV-2 virus, described in detail elsewhere [9]. In March 2020, a quick rise in SARS-CoV-2 resulted in contact restrictions and social distancing measures, and a first lockdown was implemented. Only essential public places (e.g., supermarkets and pharmacies) remained open, in compliance with strict hygiene measures (e.g., wearing face masks). In May 2020, the number of SARS-CoV-2 infections decreased, and a ‘no lockdown’ period followed until the late summer of 2020. During this period, contact restrictions and social distancing measures still applied, but cafes, restaurants, and retail stores were open. In November 2020, the SARS-CoV-2 infections increased again. Most public health measures from the first lockdown were reinstalled during this second lockdown period, which lasted until May 2021. Toward the summer of 2021, the number of infections decreased. During this second ‘no lockdown’ period, contact restrictions and social distancing measures were gradually removed.

The COVID-19 restrictions also limited the care of wound patients [10,11]. Outpatient wound clinics across Germany were closed for several weeks at the beginning of the pandemic. A cross-section survey of chronic wound patients in Germany [12] showed that the COVID-19 pandemic had a considerable impact on wound care in terms of diagnosis, hospitalization, and access to medical services. Other German studies also identified a significant impact of the pandemic on wound care [13,14,15,16].

The aim of the current study was to evaluate the impact of COVID-19 and associated lockdowns on the mood, quality of life, and immune fitness of German young adults with self-reported impaired wound healing. Research conducted before the pandemic among the same age group in The Netherlands revealed that, compared to healthy controls, young adults with self-reported impaired wound healing reported poorer mood [17], poorer sleep [18], lower quality of life [17], impaired attention and increased impulsivity [19], and a poorer immune fitness [20]. One study revealed that during the pandemic, significant lockdown effects were evident for both healthy controls and the group with self-reported impaired wound healing [11]. However, the negative effects on mood and immune fitness were significantly more pronounced among the impaired wound-healing group. For the current study, it was hypothesized that the findings would confirm and extend previous findings from The Netherlands.

## 2. Methods

An online survey was conducted among German young adults from 18 to 35 years old. Participants were recruited via email and printed flyers. The survey was administered via the online platform LimeSurvey (LimeSurvey GmbH, Hamburg, Germany) and could be completed in German or English language. Participants could enter a prize draw to win one of four 25 Euro Amazon gift cards. The study was conducted between mid-November 2021 and the end of March 2022, and informed consent was obtained from all participants. Ethics approval was obtained from the Ethics Committee of the Medical Faculty of TU Dresden (Approval code: SR-EK-8012020, date of approval: 27 September 2021). A detailed description of the study methodology and the dataset are published elsewhere [9].

Participants indicated whether or not they had experienced slow-healing wounds and/or wound infections during the past year. Based on their answer, they were allocated to (1) a control group that answered ‘no’ to both questions and (2) an IWH group that reported experiencing wound infection and/or slow-healing wounds.

Demographic data were collected, including age and sex. Attention control was assessed with the 20-item Attention Control Scale (ATTS) [21]. Items had 4 possible answers, including (1) almost never, (2) sometimes, (3) often, and (4) always. In addition to the ATTS total score, 2 subscales were computed, assessing ‘attention focusing’ and ‘attention shifting’. Mood was assessed with single-item ratings, including the items “stress”, “anxiety”, “depression”, “fatigue”, “loneliness”, “optimism”, and “happiness”. These were rated on scales ranging from 0 (absent) to 10 (extreme). The single-item scales have been validated previously [22] and have high test-retest reliability [23]. Quality of life was rated on a scale ranging from 0 (very poor) to 10 (excellent). The single-item quality of life scale is a global assessment that was validated previously against the multi-item Medical Outcomes Study Short Form-20 (MOS SF-20) and Rotterdam Symptom Check-List (RSCL) [24]. Immune fitness was assessed on a scale ranging from 0 (very poor) to 10 (excellent) [25,26]. All assessments were made for (1) ‘BP’ (the period before the COVID-19 pandemic), (2) ‘L1’ (the first lockdown period, March–May 2020), (3) ‘NL1’ (the first no-lockdown period, summer 2020), (4) ‘L2’ (the second lockdown, November 2020 to May 2021), and (5) ‘NL2’ (the second no-lockdown period, summer 2021).

Statistical analyses were conducted with SPSS (IBM Corp. Released 2013. IBM SPSS Statistics for Windows, Version 29.0. Armonk, NY, USA: IBM Corp.). Within-subject comparisons of the mood assessments of the four time points were conducted with the Related-Samples Friedman’s Two-Way Analysis of Variance by Ranks test. Bonferroni’s correction was applied, and differences were considered significant if *p* < 0.0125. Comparisons between the IWH and control group were conducted with the Independent-Samples Mann-Whitney U Test. Percentual data were compared with the N-1 Chi-squared test. Differences between the groups were considered statistically significant if *p* < 0.05.

## 3. Results

A total of n = 317 individuals participated in the study. Of them, n = 66 were allocated to the IWH group, and n = 251 to the control group. Their demographic data are summarized in Table 1.

The control group comprised significantly more women than the IWH group. The IWH group reported a significantly lower perceived immune fitness and significantly more individuals of this group had reduced immune fitness. In addition, the IWH group reported a significantly lower quality of life than the control group. The difference in sleep quality between the groups was not statistically significant.

### 3.1. Attention Control

Results on attention control are summarized in Table 2. The attention focusing score of the IHW group was significantly lower compared to the control group.

### 3.2. Mood

**Figure 1:**
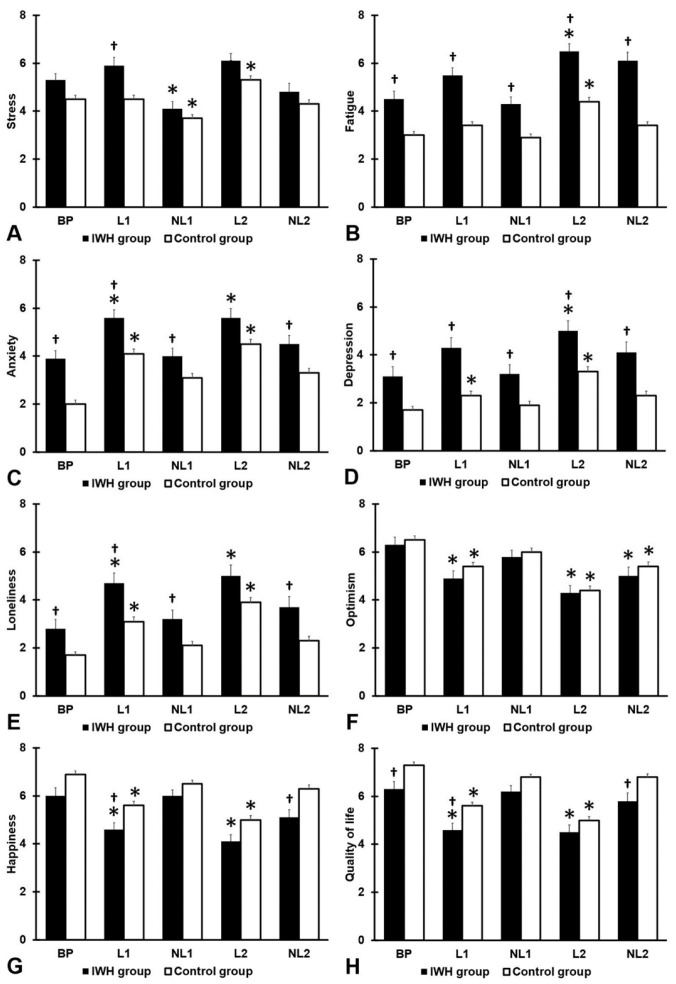
Mood and quality of life. Mean and standard error are shown for (**A**) stress, (**B**) fatigue, (**C**) anxiety, (**D**) depression, (**E**) loneliness, (**F**) optimism, (**G**) happiness, and (**H**) quality of life. Significant differences from BP (*p* < 0.0125, after Bonferroni’s correction) are indicated by *. Significant differences between the IWH group and control group (*p* < 0.010, after Bonferroni’s correction) are indicated by †. Abbreviations: BP = before the COVID-19 pandemic, L1 = first lockdown period, NL1 = first no lockdown period, L2 = second lockdown period, NL2 = second no lockdown period, IWH = impaired wound healing.

The mood and quality of life outcomes are summarized in Figure 1 and Table 3. The analysis revealed that, compared to before the pandemic, mood and quality of life were significantly poorer during the lockdown periods.

The mood effects were more pronounced in the second lockdown than in the first lockdown. During the two no-lockdown periods, mood and quality of life did not differ from before the COVID-19 pandemic, except for stress (which was significantly lower during the first no-lockdown period) and optimism (which was significantly lower during the second no-lockdown period).

### 3.3. Immune Fitness

**Figure 2:**
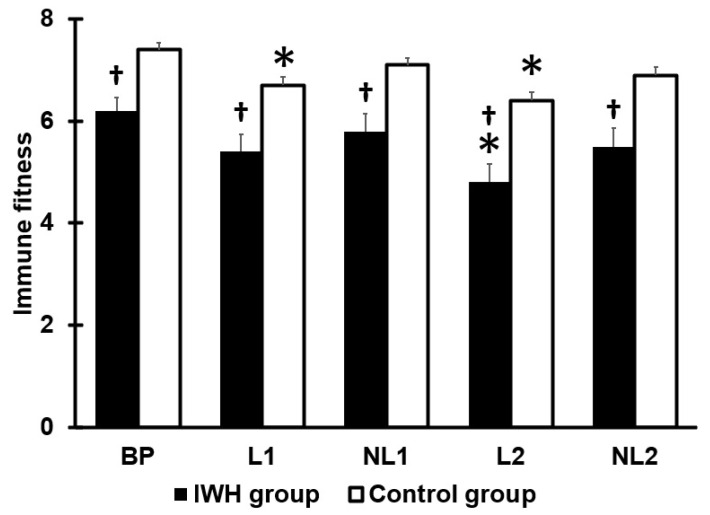
Immune fitness. Mean and standard error are shown. Significant differences from BP (*p* < 0.0125, after Bonferroni’s correction) are indicated by *. Significant differences between the IWH group and control group (*p* < 0.010, after Bonferroni’s correction) are indicated by †. Abbreviations: BP = before the COVID-19 pandemic, L1 = first lockdown period, NL1 = first no lockdown period, L2 = second lockdown period, NL2 = second no lockdown period, IWH = impaired wound healing.

Data on immune fitness is summarized in Figure 2. Compared to before the COVID-19 pandemic, immune fitness was significantly poorer during both lockdown periods for the control group, and for the IHW group, the reduction was statistically significant only for the second lockdown period. For all assessed periods, the immune fitness of the IWH group was significantly lower than that reported by the control group.

## 4. Discussion

This study in young adults in Germany confirmed that mood, quality of life, and immune fitness are poorer among individuals with self-reported impaired wound healing compared to healthy controls. It extended previous knowledge on the possible impact of attentional deficits [19] by showing that individuals with self-reported impaired wound healing have poorer attention focusing, whereas attention shifting did not differ from healthy controls. During the COVID-19 pandemic, significant negative effects were seen on mood, quality of life, and immune fitness, which were most pronounced and often statistically significant for the two lockdown periods. These findings were usually more pronounced during the second lockdown period than the first lockdown and are in line with those found in The Netherlands among young adults with self-reported impaired wound healing [11].

The current study did not investigate the causes of the observed differences in lockdown effects on mood, quality of life, and immune fitness between the group with and without self-reported impaired wound healing. It could be speculated that intermittent or poor wound treatment due to postponed care during the pandemic may be responsible for the differences. Alternatively, it may be in circumstances of psychological distress (such as a lockdown) that this may have a greater impact on individuals with impaired wound healing compared to healthy controls. Previous research in support of this hypothesis found that individuals with self-reported impaired wound healing reported significantly lower levels of mental resilience than healthy controls [17]. In addition, because individuals with impaired wound healing have a greater susceptibility to experiencing immune-related complaints [20], it could be speculated that increased fear of contracting COVID-19 could have played a role in mood changes during the pandemic. In addition, poorer immune fitness has been identified as the most important predictor of the presence and severity of COVID-19 symptoms [27]. Future studies should evaluate these possibilities to properly analyze the impact of different factors associated with poorer mood among individuals with impaired wound healing. This future research is also important beyond the context of the COVID-19 pandemic. For example, literature on patients recovering from injury or surgery revealed that positive mood and mental resilience contribute to better treatment and recovery [28,29]. In line with this, supporting positive mood and mental resilience could also significantly contribute to more successful treatment of chronic wound patients.

Limitations of this study include the fact that the data were self-reported and assessed retrospectively. Therefore, recall bias may have had an impact on the participants’ responses. Second, the classification of the participants into the impaired wound healing group versus the control group was based on self-reported data, but given the nature of the study design (an anonymous survey), this could not be verified by a formal diagnosis. Future studies should preferably be conducted in formally diagnosed patients (e.g., patients with diabetic or vascular foot ulcers). Third, the study comprised a relatively small convenience sample of young adults (18 to 35 years old) living in Germany. Therefore, it is unclear to what extent our findings can be generalized to other age groups (e.g., the elderly) or other countries where COVID-19 measures may have been different, but also the lifestyle and aspirations of young adults may be different from Germany [30,31]. The small sample size did not allow for the investigation of possible sex differences, and the age range was small. In addition, factors such as race, ethnicity, and health comorbidities were not considered. The fact that impaired wound healing was self-reported, and the fact that a convenience sample was recruited, may have resulted in an impaired wound healing group with relatively mild wound complaints and false positives. However, the fact that the current sample already shows significant differences from the control group suggests that the actual effects on mood, quality of life, and immune fitness will be even more profound in formally diagnosed chronic would patients. Future research should confirm this. Mood and quality of life were assessed with single-item ratings. While these global assessments can assess various constructs in a relatively short time, there are multiple-item scales available for both mood and quality of life. Often these scales are disease-specific and provide additional information compared to a global single-item assessment. Finally, it would be interesting for future research to contrast the current findings with other patients with other diseases for which comparable lockdown effects were investigated [32] and to evaluate the impact of comorbid diseases (e.g., diabetes).

Notwithstanding these limitations, during the COVID-19 lockdowns in Germany, significant reductions in mood, quality of life, and perceived immune functioning were reported. However, these effects were significantly more pronounced among individuals with self-reported impaired wound healing compared to healthy controls.

## Figures and Tables

**Table 1:** Demographics, health correlates, and quality of life.

	Control Group	IWH Group	*p*-Value
N	251	66	
Sex (m/f) (%)	35.5%/64.5%	21.2%/78.8%	0.028 *
Age	25.7 (4.1)	24.8 (3.9)	0.134
Immune fitness	6.9 (2.2)	5.5 (2.6)	<0.001 *
Reduced immune fitness (%)	24.90%	56.6%	<0.001 *
Sleep quality	6.7 (2.3)	6.1 (2.3)	0.105
Quality of life	6.8 (2.0)	5.8 (2.4)	0.005 *

Significant differences between the IWH and control group (*p* < 0.05) are indicated by *. Reduced immune fitness was defined as having a perceived immune fitness score < 6. Abbreviation: IWH = impaired wound healing.

**Table 2:** Attention control.

	Control Group	IWH Group	*p*-Value
ATTC total score	51.2 (7.6)	49.4 (7.9)	0.151
ATTC—Attention focusing	22.6 (4.3)	20.7 (4.2)	0.007 *
ATTC—Attention shifting	28.7 (4.7)	28.6 (4.9)	0.906

Significant differences between the IWH and control group (*p* < 0.05) are indicated by *. Abbreviations: ATTC = Attention Control Scale, IWH = impaired wound healing.

**Table 3:** Mood and quality of life.

		Mean (SD)					Overall	Pairwise Comparisons (*p*-Values)			
Assessment	Group	BP	L1	NL1	L2	NL2	*p*-Value	BP vs. L1	BP vs. NL1	BP vs. L2	BP vs. NL2
Stress	IWH	5.3 (2.0)	5.9 (2.6)	4.1 (2.3)	6.1 (2.3)	4.8 (2.6)	<0.001 *	0.120	0.004 *	0.042	0.256
	Control	4.5 (2.5)	4.5 (2.6)	3.7 (2.4)	5.3 (2.6)	4.3 (2.6)	<0.001 *	0.824	<0.001 *	<0.001 *	0.382
	*p*-value	0.024	<0.001 ^†^	0.295	0.060	0.172					
Fatigue	IWH	4.5 (2.6)	5.5 (2.3)	4.3 (2.3)	6.5 (2.4)	5.1 (2.7)	<0.001 *	0.031	0.244	<0.001 *	0.039
	Control	3.0 (2.4)	3.4 (2.5)	2.9 (2.3)	4.4 (2.7)	3.4 (2.5)	<0.001 *	0.027	0.439	<0.001 *	0.012
	*p*-value	<0.001 ^†^	<0.001 ^†^	<0.001 ^†^	<0.001 ^†^	<0.001 ^†^					
Anxiety	IWH	3.9 (2.5)	5.6 (2.6)	4.0 (2.5)	5.6 (2.9)	4.5 (2.7)	<0.001 *	<0.001 *	0.881	<0.001 *	0.310
	Control	3.0 (2.6)	4.1 (2.9)	3.1 (2.6)	4.5 (3.0)	3.3 (2.7)	<0.001 *	<0.001 *	0.496	<0.001 *	0.173
	*p*-value	0.008 ^†^	<0.001 ^†^	0.008 ^†^	0.016	0.001 ^†^					
Depression	IWH	3.1 (3.1)	4.3 (3.2)	3.2 (3.0)	5.0 (3.1)	4.1 (3.4)	<0.001 *	0.012	0.788	<0.001 *	0.049
	Control	1.7 (2.3)	2.3 (2.7)	1.9 (2.4)	3.3 (3.1)	2.3 (2.7)	<0.001 *	0.005 *	0.466	<0.001 *	0.013
	*p*-value	<0.001 ^†^	<0.001 ^†^	0.002 ^†^	<0.001 ^†^	<0.001 ^†^					
Loneliness	IWH	2.8 (2.8)	4.7 (3.1)	3.2 (2.7)	5.0 (3.4)	3.7 (3.3)	<0.001 *	<0.001 *	0.591	<0.001 *	0.073
	Control	1.7 (2.1)	3.1 (3.0)	2.1 (2.5)	3.9 (3.2)	2.3 (2.6)	<0.001 *	<0.001 *	0.388	<0.001 *	0.059
	*p*-value	0.008 ^†^	<0.001 ^†^	0.003 ^†^	0.023	0.005 ^†^					
Optimism	IWH	6.3 (2.3)	4.9 (2.3)	5.8 (2.0)	4.3 (2.2)	5.0 (2.7)	<0.001 *	0.007 *	0.370	<0.001 *	0.002 *
	Control	6.5 (2.5)	5.4 (2.4)	6.0 (2.3)	4.4 (2.6)	5.4 (2.7)	<0.001 *	<0.001 *	0.030	<0.001 *	<0.001 *
	*p*-value	0.422	0.166	0.291	0.736	0.441					
Happiness	IWH	6.0 (2.4)	4.6 (2.1)	6.0 (1.9)	4.1 (2.2)	5.1 (2.5)	<0.001 *	<0.001 *	0.952	<0.001 *	0.013
	Control	6.9 (2.2)	5.6 (2.5)	6.5 (2.2)	5.0 (2.5)	6.3 (2.4)	<0.001 *	<0.001 *	0.039	<0.001 *	0.037
	*p*-value	0.017	0.004 ^†^	0.054	0.017	0.001 ^†^					
Quality of life	IWH	6.3 (2.3)	4.6 (1.9)	6.2 (1.8)	4.5 (2.2)	5.8 (2.4)	<0.001 *	<0.001 *	0.806	<0.001 *	0.311
	Control	7.3 (1.9)	5.6 (2.4)	6.8 (1.8)	5.0 (2.4)	6.8 (2.0)	<0.001 *	<0.001 *	0.167	<0.001 *	0.145
	*p*-value	0.002 ^†^	0.006 ^^†^^	0.026	0.341	0.005 ^^†^^					

Pairwise comparisons of the differences between before the COVID-19 pandemic and the other time periods were computed if the main effect was significant (*p* < 0.05) and considered significant if *p* < 0.0125 after Bonferroni’s correction for multiple comparisons, indicated by *. Differences between the IWH and control group were considered significant if *p* < 0.010 (after Bonferroni’s correction), indicated by ^†^. Abbreviations: BP = before the COVID-19 pandemic, L1 = first lockdown period, NL1 = first no lockdown period, L2 = second lockdown period, NL2 = second no lockdown period, IWH = impaired wound healing.

## Data Availability

The data is published open access in the journal MDPI Data and available online as a supplement to reference [9].
